# Sick-listed employees with severe medically unexplained physical symptoms: burden or routine for the occupational health physician? A cross sectional study

**DOI:** 10.1186/1472-6963-10-305

**Published:** 2010-11-08

**Authors:** Rob Hoedeman, Boudien Krol, Annette H Blankenstein, Petra C Koopmans, Johan W Groothoff

**Affiliations:** 1 University Medical Center Groningen, Department of Health Sciences, University of Groningen, The Netherlands; 2 ArboNed Occupational Health Services, Utrecht, The Netherlands; 3 VU University Medical Center, Department of General Practice, EMGO Institute for Health and Care Research, Amsterdam, The Netherlands

## Abstract

**Background:**

The two primary objectives of this study were to the assess consultation load of occupational health physicians (OHPs), and their difficulties and needs with regard to their sickness certification tasks in sick-listed employees with severe medical unexplained physical symptoms (MUPS). Third objective was to determine which disease-, patient-, doctor- and practice-related factors are associated with the difficulties and needs of the OHPs.

**Methods:**

In this cross-sectional study, 43 participating OHPs from 5 group practices assessed 489 sick-listed employees with and without severe MUPS. The OHPs filled in a questionnaire about difficulties concerning sickness certification tasks, consultation time, their needs with regard to consultation with or referral to a psychiatrist or psychologist, and communication with GPs. The OHPs also completed a questionnaire about their personal characteristics.

**Results:**

OHPs only experienced task difficulties in employees with severe MUPS in relation to their communication with the treating physician. This only occured in cases in which the OHP attributed the physical symptoms to somatoform causes. If they attributed the physical symptoms to mental causes, the OHPs reported a need to consultate a psychiatrist about the diagnosis and treatment.

**Conclusions:**

OHPs experience few difficulties with their sickness certification tasks and consultation load concerning employees with severe MUPS. However, they encounter problems if the diagnostic uncertainties of the treating physician interfere with the return to work process. OHPs have a need for psychiatric expertise whenever they are uncertain about the psychiatric causes of a delayed return to work process. We recommend further training programs for OHPs. They should also have more opportunity for consultation and referral to a psychiatrist, and their communication with treating physicians should be improved.

## Background

In studies focusing on sickness absence an association has been found between medically unexplained physical symptoms (MUPS) and more frequent and prolonged sickness absence [1-4]. In sick-listed employees [5] in the Netherlands, we found a prevalence rate of 15.1% for severe MUPS (defined as a score of ≥15 on the Patient Health Questionnaire). In this sample, severe MUPS was associated with a high prevalence of depressive and anxiety disorders, distress, health anxiety and functional limitations. Given this prevalence of severe MUPS and the association with prolonged and frequent sickness absence, it can be assumed that employees with MUPS have a significant impact on the consultation load of physicians who perform sickness certification tasks.

When physicians perform sickness certification tasks and the employees symptoms are considered to be somatically unexplained, and work capacity is considered to be reduced, the risk of sickness certification is enhanced [4]. Somatisation, defined here as the attribution of the MUPS to a somatic disease and seeking medical help for these symptoms [6], contributes to a worse prognosis for recovery and to difficulties in the physician-patient relationship [7].

Norrmén et al. [8] found that physician-related factors such as long-term professional experience and working part-time resulted in more sickness certifications. No associations were found with the gender of the physician. Differences can also depend on the discipline of the physician (i.e. general practitioner, medical specialist, occupational health physician) who performs the sickness certification tasks, the work setting, and whether or not the physician has previous knowledge of the patient [9].

Shiels and Gabbay [10] found that the age of the patient and the diagnosis of a mild mental disorder (compared to all other diagnoses) increased the risk of long-term sickness absence, whereas physician-related factors did not.

Little is known about the needs of physicians who are responsible for the sickness certification of employees with MUPS. According to reviews [11,12], if there is no spontaneous recovery from MUPS, the appropriate evidence-based interventions are cognitive behavorial treatment, antidepressant medication and multidisciplinary treatment.

In the Netherlands the law states that employees who report sick have to be seen by an OHP within six weeks after they are sick-listed. The OHP establishes the diagnosis, the disabilities and the prognosis for return to work. Most visits in the Netherlands to OHPs concern consultations for sickness certification.

The Dutch health care system differentiates between consultations with curative physicians (GPs and medical specialists) who provide treatment, and consultations with the OHPs who are responsible for sickness certification and return to work, are separated. However, in practice there is an overlap in advisory tasks. It has recently become possible for OHPs to refer employees to medical specialists.

In this paper we report on data we collected to assess the difficulties and needs of OHPs in relation to sick-listed employees with and without severe MUPS, including data concerning the characteristics of the OHP. The data were collected in a prevalence study, and the employee-related characteristics have been reported in detail elsewhere [5]. The characteristics of the employees are presented in Table 1.

**Table 1:** Characteristics of sick-listed employees with and without severe MUPS*

	N = 489	PHQ 15+ * (15.1%) N = 74	PHQ 15- (84.9%) N = 415	Statistic	p-value
Female %	59.0	73.0	56.6	χ^2 ^= 6.9 df 1	0.008
Age mean (SD)	44.6 (10.0)	42.5 (9.2)	45.0 (10.0)	T(486) = 2.1	0.040
Education high/average/low %	33.1/46.6/20.3	20.0/55.7/24.3	35.5/44.8/19.6	χ^2,a ^= 4.6 df 1	0.031
White %	86.6	75.3	88.6	χ^2 ^= 9.4 df 1	0.002
Married/living together/alone %	55.9/14.1/30.0	46.6/11.0/42.5	57.7/14.7/27.6	χ^2 ^= 6.5/df 2	0.038
PHQ-15 mean (SD)	9.8 (5.4)	19.2 (2.6)	8.2 (3.9)	Z = -13.7 MW	<0.001
Duration of sick leave (days) on day of consultation (median)	123	135	121	Z = -1.2 MW	0.248
OHP diagnosis** (%)					
Mental disorder	43.1	62.2	39.8	χ^2 = ^11.9 df1	<0.001
Musculoskeletal disorder	26.8	10.8	29.6	χ^2 = ^11.4 df 1	<0.001
Other disorder	30.1	27.0	30.6	χ^2 = ^0.4 df 1	0.537
OHP attribution of the physical symptoms to somatic causes: completely/partly/not or unclear	44.3/21.6/34.1	25.4/25.4/49.3	47.9/20.8/31.3	χ^2 = ^11.9^b ^df1	0.001^ac^
Idem to psychiatric causes: completely/partly/not or unclear	12.1/17.1/70.9	25.4/26.9/47.8	9.6/15.2/75.2	χ^2 = ^21.7^b ^df 1	0.001^a^
Idem to distress: completely/partly/not or unclear	26.1/23.8/50.1	36.4/25.8/37.0	24.2/23.4/52.4	χ^2 = ^5.6^b ^df 1	0.018^a^
Idem to health anxiety: completely/partly/not or unclear	3.6/19.7/76.8	7.5/32.8/59.7	2.8/17.2/80.0	χ^2 = ^13.1^b ^df 1	0.001^a^
Idem to somatisation: completely/partly/not or unclear	5.9/23.2/70.9	6.0/29.9/64.2	5.9/22.0/72.1	χ^2 = ^1.0^b ^df 1	0.309^a^

* Severe MUPS is operationalised as a score of 15 or higher on the Patient Health Questionnaire

**) In employees who reported physical symptoms

a) X^2 ^trend test b) χ^2 ^trend test ^c ^Independent Students' t test

The present paper addresses the following research questions:

*1. *What is the difference in the OHP's consultation load due to sick-listed employees with and without severe MUPS?

*2. *Which difficulties with sickness certification do OHPs encounter, and what are their needs with regard to communication with the GP and consultation of a psychiatrist or a psychologist, concerning sick-listed employees with and without severe MUPS?

*3. *With regard to the difficulties and needs of the OHP concerning sick listed employees, with severe MUPS; with which disease-, patient-, doctor- and practice-related factors are these difficulties and needs associated?

## Methods

### Design

The present study has a cross-sectional design.

### Patients

Sick-listed employees were included in the study from April 2006 until December 2007.

### OHPs

Forty three OHPs from five group practices, covering two large occupational health services in the Netherlands, participated in this study. The five group practices were providing services to different sized organisations and different branches, and located in urban as well as rural areas. They were selected in this way in order to obtain a representative sample.

### Data-collection

During a six-week period, in the participating group practices all sick-listed employees who had an appointment with the OHP were sent a questionnaire one week before the actual consultation. The researcher (RH) collected the completed questionnaires just before the consultation with the OHP. The OHP filled in a separate questionnaire about the employee directly after the consultation. At the end of the sixth week the OHPs were asked to fill in an additional questionnaire concerning their own characteristics.

### Measures

#### Patient questionnaires

The employee was asked to fill in a questionnaire on socio-demographic variables and also questionnaires on MUPS and psychiatric co-morbidity [5]. MUPS was assessed with the somatisation sub-scale of the Patient Health Questionnaire [13] (PHQ-15). The PHQ-15 rates how much the patient has been bothered during the past month (score 0-2; not at all bothered to bothered a lot) by 15 common somatic symptoms that rarely have organic explanations. The cut-off point of 15 (severe MUPS; PHQ-15 ≥ 15) is comparable with clinically representative samples of somatoform disorders with a lower threshold than somatisation disorder [13,14];

All data on sick reports and return to work were checked by computerised registration in the two participating occupational health services.

### OHP questionnaires

#### OHP questionnaire - employee and consultation characteristics

This part of the OHP questionnaire contained questions about the presence of physical complaints in the employee, the employee's attribution of the cause of the physical complaints, and the diagnosis made by the OHP.

Consultation load was measured according to the length of the consultation in minutes.

In all cases in which the employee reported one or more physical symptoms to the OHP, ten questions were asked about the OHP's difficulties with the sickness certification tasks, with yes/no response options.

Topics were: work ability, return to work plan, activities performed by the employee to promote recovery, communication with the employee, job adjustments, communication with the employer, medical treatment provided by the treating physician, referral for further treatment, and communication with the GP.

Questions were also asked about the needs of the OHPs with regard to professional expertise concerning the diagnosis, treatment or referral to mental health professionals, and communication with the GP. with yes/no response options.

### OHP questionnaire - OHP characteristics

Each OHP filled in a questionnaire about his or her socio-demographic characteristics (gender, age), work-related characteristics (branches and organisations they served, work experience, working hours, satisfaction with the time available for consultations) and the following two questionnaires to assess burnout and work engagement of the OHP:

1) The 20-item version of the Utrecht Burnout Scale [15] (UBOS-C), which has a 3-factor structure with Crohnbachs alpha for the 3 scales above 0.70, except in some samples for the depersonalisation scale. A score higher than 2.2 for exhaustion, higher than 2.0 for cynicism and lower than 3.5 for professional efficacy, or a score higher than 2.2 for both exhaustion and cynism indicates burnout. With this questionnaire burnout can to some extent be differentiated from other mental syndromes (e.g. anxiety and depression) and that the exhaustion scale can differentiate between employees with and without burnout [16].

2) The 17-item version of the Utrecht Work Engagement Scale [17] (UWES). The UWES has a 3-factor structure relating to the factors vigor, dedication and absorption. The three factors are specific and consistent (Cronbach's alpha between 0.70 and 0.90), but have a high inter-correlation and can be used as a one dimensional construct [17]. A mean score higher than 4.67 (25^th ^percentile) for vigor indicates engagement.

### Data-analysis

The cut-off point for the MUPS score (PHQ-15) was set at 15, which according to earlier studies is an indication of clinically meaningful MUPS [13,14]. The data were dichotomized to a PHQ ≥15 group (the PHQ+ group) and a PHQ <15 group (the PHQ - group).

Difficulties the OHPs had with the sickness certification tasks were compared between the PHQ+ group and the PHQ - group. The same applied to the needs of the OHPs, with regard to communication with the treating GP, and consultation with or referral to a psychiatrist or psychologist for diagnostics, treatment, or any other reason.

For continuous variables, Independent Student's *t-*tests were used for normal distributions, and Mann-Whitney *U-*tests were used for abnormal distributions.

In a multilevel analysis, odds ratios (ORs)with 95% confidence interval (CI) were calculated for the dichotomized PHQ score, with difficulties and needs of the OHP as dependent variables and OHP as random effect. The ORs were adjusted for gender and age. A Wald score higher than 3.84 was considered to be significant. If the PHQ score was a significant predictor of difficulties or needs, a multiple logistic regression analysis was performed to determine which disease-, patient-, doctor- and practice-related variables confounded these relationships. The analyses were performed in SPSS for Windows version 15.0 and MLWIN version 2.10.

### Ethics

Ethical approval was obtained from the Medical Ethics Committee of the University Medical Center in Groningen, who informed us that ethical clearance was not required because only self-report questionnaires were used and the results are reported at group level.

## Results

We achieved a 97.2% response from the OHPs with regard to their assessments of employees who had filled in their questionnaires and consulted the OHP for sickness certification.

The response rate from the OHPs with regard to their characteristics was 97.7%, with an equal distribution between male and female OHPs. The large majority of the OHPs considered it their task to search for psychosocial explanations for symptoms and, if present, to explain these to the sick-listed employees.

None of the OHPs reported burnout. According to the UWES scores, there was at least average engagement, although the scores did not meet the criteria for real engagement (score above 4.67; 25^th ^percentile of reference value). See Table 2.

**Table 2:** Characteristics of OHPs

Group practice number	1	2	3	4	5	Total
No of OHPs	13	7	6	8	9	43
Size of organisations	>500 employees	>500 employees	<75 employees	<75 and 75-500 employees	>500 employees	
Main branches	Public services, education and health service	Government	All types	All types	Public services, financial services and local government	
Urbanisation	Urban	Urban	Rural	Urban	Mixed	
Age mean (SD)	48.7 (6.5)	45.1 (5.6)	42.2 (9.7)	46.3 (7.4)	47.8 (5.3)	46.5 (6.9)
% female	30.8	71.4	50.0	50.0	87.5	54.8
Work experience (years) (SD)	13.4 (6.2)	13.4 (2.4)	9.5 (7.7)	11.1 (4.3)	12.3 (3.4)	12.2 (5.1)
% working hours of fulltime mean (SD)	89.2 (13.2)	76.4 (13.8)	91.7 (16.0)	77.5 (16.7)	81.3 (25.3)	83.7 (17.4)
% not (at all) satisfied with time available for consultations	15.4	28.6	16.7	62.5	12.5	26.2
Emotional exhaustion mean (SD)	1.2 (0.7)	1.8 (1.4)	1.6 (1.1)	1.6 (1.3)	1.9 (0.8)	1.6 (1.0)
Distance (UBOS) mean (SD)	0.6 (0.5)	0.9 (0.9)	1.1 (0.8)	1.0 (0.5)	0.9 (0.4)	0.9 (0.6)
Competences (UBOS) mean (SD)	4.7 (0.5)	4.8 (0.8)	4.1 (0.9)	4.8 (0.7)	4.5 (0.6)	4.6 (0.7)
Engagement (UWES) Mean (SD)	4.5 (0.6)	4.4 (0.5)	3.3 (1.0)	3.8 (1.0)	3.7 (0.6)	4.1 (0.8)

With regard to the consultation load, the OHPs did not need more time for employees with severe MUPS (25.1 minutes, standard deviation 7.7) than for employees with less severe MUPS (23.8 minutes, SD 8.6), p = 0.266.

The OHPs experienced no specific difficulties with most of the sickness certification tasks. They only experienced task difficulties concerning employees with severe MUPS in their communication with the treating physician. The OHPs only reported needs in employees with severe MUPS for the expertise of a psychiatrist. See Table 3.

**Table 3:** Prevalence of OHP tasks and needs in sick-listed employees with high and low levels of MUPS

	Percentages in PHQ+/PHQ- groups	OR (95% CI)†
**Task difficulties OHPs**:		
Indicating workability	15.9/10.8	1.48 (0.64-3.42)
Plan for return to work	17.6/11.1	1.46 (0.64-3.29)
Recovery activities employee	14.7/13.8	0.87 (0.36-2.09)
Comunication with employee	11.8/6.2	1.91 (0.76-4.81)
Job (place) adjustments	8.8/6.8	0.90 (0.29-2.87)
Communication between employer and employee	2.9/4.9	0.56 (0.13-2.51)
Communication with employer	7.4/5.1	1.81 (0.63-5.23)
Treatment provided by the attending physician	16.2/7.9	2.00 (0.78-5.10)
Referral for treatment	4.4/1.6	3.00 (0.70-12.92)
Communication with attending physician	10.3/2.4	5.42 (1.82-16.21)**
		
**Needs of OHPs**:		
Expertise of a psychiatrist		5.65 (2.32-13.78)**
- Diagnostic	9.0/2.4	1.36 (0.73-2.52)
- Treatment	7.5/1.6	2.08 (0.92-4.72)
- Other	3.0/1.1	
Expertise of a psychologist		
- Diagnostic	6.1/2.5	
- Treatment	16.7/13.4	
- Other	7.6/6.0	
Communication with GP		
- Diagnostic	11.8/2.2	
- Treatment	8.8/7.3	
- Other	0.0/1.4	

† Adjusted for gender and age with occupational physician as random effect

* p < 0.05

** p < 0.01

With regard to the OHPs' task difficulties concerning communication with the treating physician, the logistic regression analysis showed that the confounders were: the OHPs' attribution of the physical symptoms to somatoform causes and age. See Table 4.

**Table 4:** Logistic regression on the need of OHP for communication with treating physician

	B	SE	Wald	**p-value**.	OR (95%CI)
Comorbidity: depressive disorder	0.78	0.94	0.69	0.407	2.18 (0.35-13.67)
Comorbidity: anxiety or panic disorder	0.61	0.90	0.45	0.500	1.83 (0.32-10.64)
Distress (4DSQ)	-0.85	0.93	0.84	0.359	0.43 (0.07-2.63)
PHQ +	2.22	0.83	7.22	0.007	9.20 (1.82-46.46) **
Gender (female) employee	0.49	0.72	0.47	0.493	1.64 (0.40-6.72)
Age employee	0.09	0.04	4.79	0.029	1.09 (1.01-1.18) *
Emotional exhaustion OHP (UBOS-C)	0.33	0.29	1.22	0.269	1.39 (0.78-2.47)
Engagement OHP (UWES)	0.73	0.47	2.43	0.119	2.07 (0.83-5.17)
Somatic attribution OHP	0.76	0.81	0.90	0.343	2.15 (0.44-10.41)
Somatoform attribution OHP	2.17	0.77	7.92	0.005	8.80 (1.94-39.98) **
Mental attribution OHP	0.11	1.03	0.01	0.915	1.12 (0.15-8.44)
Duration sickness absence (per 10 days)	-0.01	0.02	0.52	0.469	0.99 (0.95-1.02)
Physical functioning (SF36)	0.04	0.02	2.87	0.090	1.04 (0.99-1.08)
Mental functioning (SF36)	-0.02	0.02	0.64	0.423	0.98 (0.94-1.03)

The table shows the odd ratio's (Ors) and their 95% confidence interval (CI) as well as the Wald statistics calculates as (B/SE)^2 ^in which estimate B represents the effect of the predictor on the log odds of the outcome after adjusting for all other covariates in the model and standard error (SE) the variability of this effect

* p ≤ 0.05

** p ≤ 0.01

With regard to the needs of the OHP to obtain information about diagnosis and treatment from a psychiatrist, the logistic regression analysis showed that the OHP's attribution of the physical symptoms to mental causes was a very strong confounder, because all OHPs who reported a need for consultation with a psychiatrist (n = 30) attributed the symptoms to mental causes. Due to this very strong association a model could not be constructed. In a model without the OHPs' attribution, the OHP's need was associated with the duration of the sickness absence (OR 1.02 [1.00-1.04], Wald 4.76), and not with psychiatric comorbidity.

## Discussion

With regard to the consultation load and most of the sickness certification tasks, the OHPs experienced no difference between consultations with sick-listed employees with severe MUPS and consultations with employees with less severe MUPS. Our findings are remarkable, because employees with severe MUPS present more psychiatric co-morbidity and have more functional limitations, as has been demonstrated in many other studies [5,18,19] Barsky et al. [19] found that MUPS were associated with functional limitations and more medical consumption, and psychiatric co-morbidity did not add to this effect. Furthermore, it is remarkable that the OHPs experienced no difficulties in their communication with employees with severe MUPS, unlike the treating physicians [6]. This can be explained, at least partly, by the fact that OHPs in the Netherlands have no treatment tasks, and therefore they are not often pressurised by employees in this respect [20].

With regard to consultation load and sickness certification tasks, our findings suggest that OHPs manage sick-listed employees with severe MUPS in the same way as they manage sick-listed employees with less severe forms of MUPS.

According to our findings, the most important difficulties that the OHPs experienced were associated with their communication with the treating physician. This is associated with the OHPs' attribution of the physical symptoms to somatoform causes, but from our data the exact nature of these difficulties is unclear. However, difficulties associated with certification tasks can be imagined when employee and/or the treating physicians are not convinced of the somatoform nature of the physical symptoms. Return to work can be delayed if the employee is awaiting the results of diagnostic tests, or is convinced that his/her limitations are the results of a somatic disease with limitations that will last until the disease has been treated adequately.

OHPs report the need for psychiatric expertise when they attribute physical symptoms to psychiatric causes. Contrary to what could be expected, it is not the psychiatric comorbidity that is associated with this need of the OHP. This is an indication that the OHP has diagnostic uncertainties.

A major problem is that employees with severe MUPS remain unrecognized. Thus, the group with the lowest recovery rate and the most functional limitations [5] is missed, because more physical symptoms indicate a worse prognosis [13,21]. There are evidence-based guidelines for the management of patients with chronic fatigue and fibromyalgia [22,23], and one guideline in the Netherlands for medically unexplained physical symptoms [24]. These guidelines emphasize that it is important to rule out somatic and psychiatric causes of the physical symptoms, because the prognosis depends on adequate treatment of these causes. If these causes are not present, the physician should stimulate the patient to stay active in order to prevent unnecessary inactivity, social isolation and job-loss. Guidelines for aspecific back symptoms [25], which can also be seen as a form of MUPS [11], promote a time-contingent return to work process to prevent medicalisation and lasting disabilities. Shiels and Gabbay [10] found that, with regard to sickness certification, the diagnosis is of more importance for the risk of long-term sickness than OHP-related factors. In combination with the under-recognition of severe MUPS, it is relevant to try to achieve more diagnostic accuracy, and this is an important starting point for solving this problem in the field of occupational health.

### Strengths and limitations

To our knowledge, our study is the first survey of the impact of sick-listed employees with severe MUPS on OHPs. A strength of the study is that the 43 OHPs were recruited from 5 group practices associated with 2 large occupational health services distributed throughout the country, serving urban and rural populations in different branches and different sized organisations. Another strong point is that the information about MUPS and related aspects was gathered from OHPs and employees by means of validated questionnaires.

However, due to the cross-sectional design of the study, no conclusions can be drawn with regard to causal relationships in our findings.

Self-report questionnaires for the employees and the OHPs were used to address our main research questions. No additional medical check on the MUPS was carried out, and the only diagnoses were those made by the OHPs. As reported elsewhere, we used validated questionnaires, which have high a correlation with somatisation, depression and anxiety diagnoses in primary care and clinical practice [14]. However, we did not ask the OHPs directly whether they diagnosed the symptoms as MUPS, in order to avoid bias in their diagnosis, and because this concept is not well known. This information is therefore missing.

There was no qualitative analysis of the OHP's answers with regard to diagnosis (e.g. when and how they consider the diagnosis of MUPS), task difficulties (e.g. what OHPs consider to be their task with regard to treatment) and their own characteristics (e.g. the differences in opinion among OHPs with regard to their tasks, and between OHPs with varying levels of burnout and engagement).

### Implications for practice

Our results indicate that OHPs should be trained in how to diagnose severe MUPS, for two functions: firstly, ruling out somatic and psychiatric causes helps to rule out these disorders which can delay the return to work process. Secondly: the diagnosis of severe MUPS makes it possible to explain to employees why they have severe limitations, on the one hand, and on the other hand why they should increase their activities and continue time-contingent return to work. When improved functioning and return to work are achieved, no additional steps are required.

If uncertainty about the diagnosis and adequate treatment remains, with no improvement in the employees with severe MUPS, OHPs should have easy access to psychiatric expertise and/or psychological treatment. Research shows that liaison consultation of a psychiatrist is helpful [26,27]. For patients with lasting MUPS, if motivated, cognitive behavioral therapy provided by a psychologist is the recommended evidence-based treatment [11,12]. Multidisciplinary treatment is indicated for employees with long-lasting non-specific back and neck problems or other forms of MUPS [11].

Although in the Netherlands many projects have resulted in an improvement in communication between the OHP and the GP, there is still a need for better communication and agreement [28]. As sick-listed employees with severe MUPS are at increased risk for lasting disabilities and health-related jobloss, their OHPs should make extra effort to prevent this as much as possible [5]. Treating physicians have difficulties with regard to increased medical consumption and communicating with these patients. Hopefully, the results of this study will stimulate the boards of especially OHPs and GPs to make extra efforts to achieve better communication concerning patients with severe MUPS.

OHPs and GPs must focus more on the functioning of employees with severe MUPS. This is in accordance with the decision made in the United Kingdom for GPs to write 'fit for work notes' instead of sick notes [29,30]. This was done because employees with common mental disorders are helped more by returning to (adjusted) work as soon as they can, than by remaining inactive. This is also in line with the existing evidence concerning effective methods of treatment for patients with severe MUPS [11,12]: cognitive behavorial treatment, graded activity, and communication and casement rules. Cognitive behavorial treatment [31] helps patients with severe MUPS not by focusing on the (somatic) causes of their symptoms, but on helping them to handle consequences such as inactivity, isolation, etc. Graded activity focuses on a stepwise increase in activity. Communication and casement rules in consultation letters focus on empathy from the treating physician, explaining the findings, and preventing medicalisation, and this policy also results in improvement in physical functioning [12].

### Implications for research

First, in order to formulate criteria for interventions, longitudinal research is needed to investigate the determinants of return to work in employees with severe MUPS, including their psychiatric co-morbidity and the work characteristics.

Additional research is needed to find out how the diagnostic competence of OHPs can be improved. In our opinion, the first step would be to train OHPs (in diagnosis, guidelines and communication) and provide them with diagnostic tools, such as the PHQ. In a second step the quality of the diagnosis can be improved by increasing the possibilities for OHPs to consult a psychiatrist and improving communication with GPs and other attending physicians. Finally, more research is needed to determine how these steps can be made feasible to occupational health care. A good example from primary care is the provision of collaborative care for depressed patients [32]. Qualitative research will also be useful to identify what are the needs of the sick-listed employee with MUPS, in terms of symptoms, functioning, and return to work.

### Final conclusions

MUPS and associated limitations in functioning lead to a few, but important task difficulties for OHPs: they have diagnostic uncertainty and experience interference in their tasks from the diagnostic activities and treatment provided by treating physicians. Better recognition of severe MUPS and combining improvement of functioning and the return to work process with the diagnostic and treatment activities of the the treating physicians, are aspects that can be improved. There is a need for further training of OHPs, better communication with the GP, and easier access to the expertise of psychiatrists, psychologists and multidisciplinary treatment.

## Competing interests

The authors declare that they have no competing interests.

## Authors' contributions

RH, BK, NB en JWG contributed to the design. RH collected the data. PK carried out the data analysis and gave methodological advice. All authors participated in the data-interpretation. RH wrote the manuscript. All authors revised the manuscript for important intellectual content. All authors have read and approved the final manuscript.

## Pre-publication history

The pre-publication history for this paper can be accessed here:

http://www.biomedcentral.com/1472-6963/10/305/prepub
